# Inhaled PGE_1_ in neonates with hypoxemic respiratory failure: two pilot feasibility randomized clinical trials

**DOI:** 10.1186/1745-6215-15-486

**Published:** 2014-12-12

**Authors:** Beena G Sood, Martin Keszler, Meena Garg, Jonathan M Klein, Robin Ohls, Namasivayam Ambalavanan, C Michael Cotten, Monica Malian, Pablo J Sanchez, Satyan Lakshminrusimha, Leif D Nelin, Krisa P Van Meurs, Rebecca Bara, Shampa Saha, Abhik Das, Dennis Wallace, Rosemary D Higgins, Seetha Shankaran, for the Eunice Kennedy Shriver National Institute of Child Health and Human Development Neonatal Research Network

**Affiliations:** Department of Pediatrics, Children’s Hospital of Michigan, 3901 Beaubien Blvd., 4H42, Detroit, MI 48201 USA; Department of Pediatrics, Women and Infants Hospital, Brown University, 101 Dudley Street, Providence, RI 0290 USA; Department of Pediatrics, University of California, 10833 Le Conte Avenue, Room B2-375 MDCC, Los Angeles, CA 90095 USA; Department of Pediatrics, University of Iowa, 200 Hawkins Drive, Iowa City, IA 52242 USA; MSC10 5590 1, University of New Mexico Health Sciences Center, Albuquerque, NM 87131-0001 USA; Division of Neonatology, University of Alabama at Birmingham, 176F Suite 9380 619 South 19th St, Birmingham, AL 35249-7335 UK; Department of Pediatrics, Duke University, 2424 Erwin Road Suite 504, Durham, NC 27705 USA; Department of Pediatrics, University of Texas Southwestern Medical Center, 5323 Harry Hines Boulevard, Dallas, TX 75390 USA; Department of Pediatrics, University of Buffalo, 219 Bryant Street, Buffalo, NY 14222 USA; Department of Pediatrics, The Ohio State University and Nationwide Children’s Hospital, 700 Children’s Drive, W203, Columbus, OH 43205 USA; Department of Pediatrics, Division of Neonatal and Developmental Medicine, Stanford University School of Medicine and Lucile Packard Children’s Hospital, 750 Welch Road, Suite 315, Palo Alto, CA 94304 USA; Social, Statistical and Environmental Sciences Unit, RTI International, Research Triangle Park, NC USA; Social, Statistical and Environmental Sciences Unit, RTI International, 6110 Executive Blvd., Suite 902, Rockville, MD 20852-3903 USA; Eunice Kennedy Shriver National Institute of Child Health and Human Development, National Institutes of Health, Rm 4B03, 6100 Executive Blvd., MSC 7510, Bethesda, MD 20892-7510 USA

**Keywords:** Hypoxemic respiratory failure, Neonatal, Pulmonary hypertension, Aerosols, Nebulizers, Prostaglandins, Clinical trial

## Abstract

**Background:**

Inhaled nitric oxide (INO), a selective pulmonary vasodilator, has revolutionized the treatment of neonatal hypoxemic respiratory failure (NHRF). However, there is lack of sustained improvement in 30 to 46% of infants. Aerosolized prostaglandins I_2_ (PGI_2_) and E_1_ (PGE_1_) have been reported to be effective selective pulmonary vasodilators. The objective of this study was to evaluate the feasibility of a randomized controlled trial (RCT) of inhaled PGE_1_ (IPGE_1_) in NHRF.

**Methods:**

Two pilot multicenter phase II RCTs are included in this report. In the first pilot, late preterm and term neonates with NHRF, who had an oxygenation index (OI) of ≥15 and <25 on two arterial blood gases and had not previously received INO, were randomly assigned to receive two doses of IPGE_1_ (300 and 150 ng/kg/min) or placebo. The primary outcome was the enrollment of 50 infants in six to nine months at 10 sites. The first pilot was halted after four months for failure to enroll a single infant. The most common cause for non-enrollment was prior initiation of INO. In a re-designed second pilot, co-administration of IPGE_1_ and INO was permitted. Infants with suboptimal response to INO received either aerosolized saline or IPGE_1_ at a low (150 ng/kg/min) or high dose (300 ng/kg/min) for a maximum duration of 72 hours. The primary outcome was the recruitment of an adequate number of patients (n = 50) in a nine-month-period, with fewer than 20% protocol violations.

**Results:**

No infants were enrolled in the first pilot. Seven patients were enrolled in the second pilot; three in the control, two in the low-dose IPGE_1_, and two in the high-dose IPGE_1_ groups. The study was halted for recruitment futility after approximately six months as enrollment targets were not met. No serious adverse events, one minor protocol deviation and one pharmacy protocol violation were reported.

**Conclusions:**

These two pilot RCTs failed to recruit adequate eligible newborns with NHRF. Complex management RCTs of novel therapies for persistent pulmonary hypertension of the newborn (PPHN) may require novel study designs and a longer period of time from study approval to commencement of enrollment.

**Trial registration: ClinicalTrials.gov:**

Pilot one: NCT number: 00598429 registered on 10 January 2008. Last updated: 3 February 2011.

Pilot two: NCT number: 01467076 17 October 2011. Last updated: 13 February 2013.

**Electronic supplementary material:**

The online version of this article (doi:10.1186/1745-6215-15-486) contains supplementary material, which is available to authorized users.

## Background

Neonatal hypoxemic respiratory failure (NHRF) is usually associated with widespread vasoconstriction of the pulmonary microvasculature, giving rise to intra- and extra-pulmonary shunting and profound hypoxemia. Due to the delay in the physiologic fall in pulmonary vascular resistance, it is also known as persistent pulmonary hypertension of the newborn (PPHN). The goal of therapy is to decrease the regional pulmonary vascular resistance of ventilated lung areas, thus decreasing intrapulmonary shunting and selectively reducing the pulmonary-artery pressure without causing systemic vasodilation. Intravenously administered vasodilators lack pulmonary selectivity leading to systemic side effects. Inhaled nitric oxide (INO), a selective pulmonary vasodilator, has revolutionized the treatment of respiratory failure in the newborn. However, there is lack of sustained improvement in 30 to 46% of infants [1–4]. Moreover, INO requires specialized delivery systems, making the treatment expensive.

Aerosolized prostaglandins I_2_ (PGI_2_) and E_1_ (PGE_1_) have been reported to be effective selective pulmonary vasodilators in animals, adults, and preterm and term newborns [5–15]. Compared to PGI_2_, PGE_1_ has a shorter half-life, lower acid dissociation constant (pKa, 6.3 versus 10.5), bronchodilator action, and anti-proliferative and anti-inflammatory effects on the alveolar, interstitial, and vascular spaces of the lung [10, 16–20]. In addition, PGE_1_ is readily available in pharmacies of hospitals with neonatal services and has a proven safety record from its intravenous use in ductal-dependent cardiac anomalies. Prostaglandin nebulization can be performed without the sophisticated technical equipment needed for INO and hence is less expensive. Prostaglandins and nitric oxide relax the vascular smooth muscle through two different second-messenger systems; therefore, in combination, INO and inhaled PGE_1_ (IPGE_1_) may have a synergistic effect [21].

We reported the safety and feasibility of short-term administration of IPGE_1_ in an un-blinded phase I/II dose-escalation single center study [12] suggesting the need for placebo-controlled randomized studies to establish the efficacy and safety of this drug for NHRF. In that study, four doses ranging from 25 to 300 ng/kg/min were tested for a maximum duration of three hours. The purpose of the two studies included in this report was to determine the feasibility of conducting a future large randomized control trial (RCT) to evaluate the safety and efficacy of prolonged IPGE_1_ at high-volume level III to IV neonatal intensive care units (NICUs) within the National Institute of Child Health and Human Development (NICHD) Neonatal Research Network (NRN).

## Methods

### Pilot one: a randomized clinical trial of inhaled PGE_1_ in neonatal hypoxemic respiratory failure

Following the availability of INO for the treatment of NHRF, several interventional clinical trials for NHRF were halted prematurely for lack of enrollment [22–25]. Therefore, we sought to quantify the number of potentially eligible patients and perform a pilot trial before embarking upon a RCT of IPGE_1_. Late preterm and term infants with NHRF who had an oxygenation index (OI) (OI = mean airway pressure (MAP) × fractional inspired oxygen concentration (FiO_2_) × 100/arterial oxygen tension (P_a_O_2_)) ≥15 and <25 on two arterial blood gases (ABGs) and had not previously received INO were eligible for the study. Infants would be randomly assigned to receive two doses of IPGE_1_ (300 and 150 ng/kg/min) or placebo over a maximum duration of 72 hours. The primary outcome was the ability to enroll 50 infants in six to nine months at 10 sites, without excessive protocol violations or adverse effects. Secondary outcome variables included the definition of optimal dose and duration of treatment with the study aerosol, the need for INO or extra-corporeal membrane oxygenation (ECMO) mortality, and duration of mechanical ventilation, O_2_ administration, and INO administration.

The research study was approved by the institutional review boards (IRBs) of all participating sites (see Additional file 1). The first pilot RCT was halted after four months for failure to enroll any infants. The most common cause for inability to enroll infants was the rapidly changing condition of the infants, resulting in prior initiation of INO in transport or soon after admission. The next most frequent causes of inability to recruit infants were lack of an arterial line and hypothermia treatment for neonatal encephalopathy, both of which were exclusion criteria.

### Pilot two: a randomized clinical trial of inhaled PGE_1_ in neonates with sub-optimal response to inhaled nitric oxide

After evaluating the causes for lack of enrollment in the first pilot, we determined that co-administration of IPGE_1_ and INO could potentially have a substantial positive impact on study feasibility, while allowing us to test for synergy between INO and IPGE_1_. In addition, we elected to include infants receiving therapeutic hypothermia, as this was now standard therapy in participating centers.

In order to design and meaningfully interpret the data from the second pilot RCT in a manner that would be most helpful for designing the main trial, we created a definition of the primary outcome and conducted sample size calculations for the main trial. Using the composite outcome of need for ECMO or death as the most concrete clinically meaningful outcome for the future main trial, a sample size of 149 patients per group would be required to detect a 16% absolute risk reduction in the composite outcome of ECMO or death from 50% in the control arm to 34% in the study arm, with α of 0.05, a power of 80%, and a two-tailed test. To get an estimate of the number of infants in the NRN sites that might be eligible for the main trial, we queried all NRN sites. Between 2009 and 2010, 775 infants received INO for NHRF in 17 sites in the NRN; 30 to 35% met the composite outcome of death or ECMO and 10 to 13% died without receiving ECMO. Based on these numbers, it would be feasible to perform the main trial of IPGE_1_ in NHRF over three years or less.

Study eligibility was modified in the second pilot to include infants who had a suboptimal response to INO defined as an OI of ≥15 on any two arterial blood gases, obtained 15 minutes to 12 hours apart, and during the first 72 hours of INO use. The research study was approved by the IRBs of all participating sites and informed parental consent was obtained for all enrolled infants once eligibility was confirmed and before institution of study intervention.

#### Subjects

Late preterm (34 0/7 to 36 6/7 weeks’ gestation) and term infants at less than or equal to seven days postnatal age, undergoing conventional ventilation (CNV) or high-frequency oscillatory ventilation (HFOV) for NHRF (including perinatal aspiration syndrome, suspected or proven pneumonia or sepsis, respiratory distress syndrome, idiopathic PPHN, or suspected pulmonary hypoplasia), and with a suboptimal response to INO were eligible. An indwelling arterial line was a requisite for study participation, as was informed parental consent. Exclusion criteria included the decision not to provide full treatment, a known structural congenital heart disease except patent ductus arteriosus and atrial or ventricular level shunts, congenital diaphragmatic hernia, and thrombocytopenia (platelet count <80,000/μl) unresponsive to platelet transfusion.

#### Study procedures

There were three arms to the study: low-dose IPGE_1_ (150 ng/kg/min), high-dose IPGE_1_ (300 ng/kg/min), and placebo (aerosolized saline). The two doses of IPGE_1_ were selected on the basis of the results of the pre-clinical toxicity study and the phase I/II open label study of IPGE_1_ in NHRF [12]. Eligible infants were centrally randomized by telephone using permuted block randomization. The IPGE_1_ was double-masked. Only the participating center’s pharmacist was aware of the infant’s assigned group.

**Figure 1:**
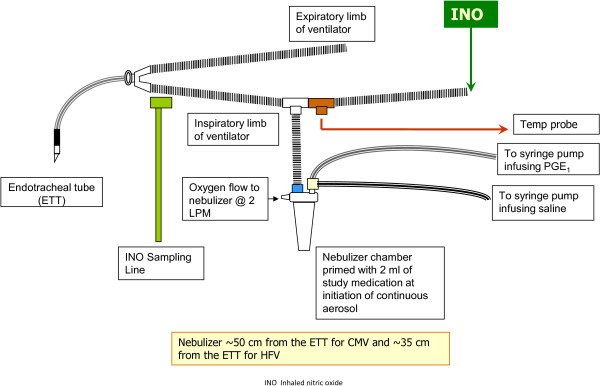
**Diagram of nebulizer setup in neonatal ventilator circuit.** Figure legend: ETT, endotracheal tube; INO, inhaled nitric oxide; CMV, conventional mechanical ventilation; HFV, high frequency ventilation; IPGE_1,_ inhaled PGE_1_ (Pfizer, New York, NY, USA).

PGE_1_ solution for aerosolization was prepared from synthetic PGE_1_ (Prostin VR (Alprostadil) 500 μg/ml in 1 ml dehydrated ethanol, Pfizer, New York, NY, USA) by dilution in sterile 0.9% saline (Sodium Chloride 0.9% Injection USP, Hospira Inc., Lake Forest, IL, USA). Fresh solutions were prepared every 24 hours. Aerosol administration began with a PGE_1_ dosage of either 300 or 150 ng/kg/min diluted in 4 ml preservative-free sterile normal saline/hr in study patients, and 4 ml sterile normal saline/hr in control patients. The study medication was delivered using a syringe pump into the nebulizer chamber of the MiniHeart low flow jet nebulizer (WestMed Inc., Tucson, Arizona, United States) (Figure 1). The nebulizer was placed approximately 50 cm from the endotracheal tube for CNV, and approximately 35 cm from the endotracheal tube in HFOV, based on bench studies to determine the emitted dose [26]. The nebulizer chamber was primed with 2 ml of study medication at study aerosol initiation. For weaning purposes, normal saline was administered using a second syringe pump with a Y-connection into the nebulizer chamber with increasing dose as the study drug aerosol dose was decreased, such that the total volume delivered was always 4 ml/hour. During aerosol administration, FiO_2_ delivered in the gas flow through the nebulizer was matched to that delivered in the ventilator circuit.

Conventional management including ventilation, hemodynamic support, and sedation were optimized prior to randomization as per usual care at site. Treatment with INO was recommended if an OI ≥25 was documented on two consecutive ABGs at least 15 minutes apart; however, depending on institutional practice, treatment with INO could be instituted at an OI <25 [4, 25]. It was recommended that an echocardiogram and cranial sonogram be obtained prior to, or soon after, the initiation of INO to rule out structural heart lesions, establish the presence of pulmonary hypertension, and evaluate for intracranial hemorrhage.

#### Response to study medication

Arterial blood gas analysis was performed 60 ± 15 minutes after study aerosol initiation. Response was defined on the basis of an increase in P_a_O_2_ (mmHg) above baseline after 60 ± 15 minutes exposure to the study aerosol as full (≥20), partial (10 to 19) or no (<10) increase in P_a_O_2_. All infants were continued on the assigned study aerosol regardless of initial response until they weaned off, met treatment failure criteria, or completed 72 hours of study aerosol administration. Arterial blood gases were drawn at four ± two hours and every 12 ± two hours thereafter. Additional blood gases were obtained as clinically indicated. After the initial 60 ± 15 minutes observation period, therapeutic decisions were left to the clinical team.

#### Primary outcome

The primary aim of the study was the ability to recruit an adequate number of patients (n = 50) in a nine-month-period without excessive (more than 20%) protocol violations.

#### Secondary outcomes

Secondary outcomes included improvement in PaO_2_ and OI at 60 ± 15 minutes and four ± two hours after study aerosol initiation; need for ECMO; death; combined outcome of need for ECMO or death in the first 120 days of life; need for supplemental O_2_ at 28 days of life; duration of INO, mechanical ventilation, supplemental oxygen, and hospitalization; and occurrence of intracerebral hemorrhage, intraventricular hemorrhage, periventricular hemorrhagic infarction, cystic leukomalacia, areas of low attenuation in the white matter (edema or ischemia), atrophy, and ventriculomegaly.

#### Treatment failure criteria

Treatment failure was defined as an acute deterioration on initiation of, or during administration of, the study aerosol with an absolute fall in pulse oximeter O_2_ saturation (SPO_2_) by more than 10% for over 10 minutes, or an absolute SPO_2_ of less than 85%. Mechanical causes, including pneumothorax or plugged or malpositioned endotracheal tube (ETT) needed to be excluded. It was recommended that ECMO be considered in infants with an OI >40, or an alveolar arterial PaO_2_ gradient >620 mmHg for eight hours, a PaO_2_ < 40 to 50 mmHg for two to four hours, or if institutional guidelines were met.

#### Weaning of study aerosol

A weaning algorithm was proposed for the study aerosol to avoid a potential drop in P_a_O_2_. For any given weaning time point, the dose was halved and weaning was attempted only if the P_a_O_2_ was >60 torr. The first weaning attempt was at 12 ± two hours. Further weaning attempts were mandated at 12 ± two hour intervals. Total duration of study aerosol administration could not exceed 72 hours. Weaning to the third dose had to be accomplished by 48 hours on study aerosol.

#### Weaning of ventilator during study

Once the study aerosol was initiated, attempts to wean INO dose and/or ventilator settings were protocol-driven and every 12 hours or more frequently as deemed appropriate by the clinical team.

#### Monitoring of study aerosol administration

Enrolled infants were monitored continuously throughout study aerosol administration for possible adverse effects of PGE_1_, including hyperthermia (>38°C), bradycardia (heart rate <70/min, arrhythmia, hypotension, seizures, bleeding tendency, pulmonary hemorrhage, diarrhea, and seizures. Study aerosol was weaned if hypotension or arrhythmia persisted despite maximal therapy. In addition, all newborns enrolled in the study were clinically evaluated for signs and symptoms of patent ductus arteriosus (PDA at well-defined time points (three ± one and seven ± one days after discontinuation of the study medication and within one week before discharge).

#### Protocol violations

Protocol violations included: wrong study aerosol administered; wean to dose three not completed by 48 +/- four hours; study aerosol given for more than 72 +/- four hours; and unmasking of the study aerosol.

#### Sample size

The pilot RCTs were primarily designed to evaluate the feasibility and safety of prolonged IPGE_1_ administration and determination of optimal dose in 50 patients recruited at high-volume sites within the NRN. Comparison of the combined IPGE_1_ groups (n = 100) with the control group (n = 50) would have a power of 76.9% to detect a difference of 16% absolute risk reduction in the composite outcome of ECMO or death, from 50% in the control arm to 34% in the study arm, with α of 0.25 and a two-tailed test. The higher type I error rate could erroneously lead to a conclusion of efficacy of IPGE_1_ but would not increase the risk of missing an efficacious treatment [27–29]. While statistically significant evidence of treatment efficacy was unlikely in these small pilot trials, we expected a relative risk of 67% (range: 50 to 80%) for the treatment compared to the control arm for the combined outcome of need for ECMO or death in 50 patients recruited over a time period of nine months to be able to justify embarking upon the full-scale RCT.

#### Data Safety Monitoring Committee

The NICHD NRN Data Safety Monitoring Committee (DSMC) monitored the progress of the trial for feasibility and safety. Feasibility was defined *a priori* as the ability to recruit at least four subjects per month over any rolling three-month-period once 50% of the participating sites had IRB approval; and six subjects per month over any rolling three-month-period once 75% of the participating sites had IRB approval. Given those accrual targets, if fewer than 20 subjects had been enrolled in the six-month-period after at least 75% of the sites had achieved IRB approval, the DSMC could recommend that the trial be stopped for futility.

## Results

**Figure 2:**
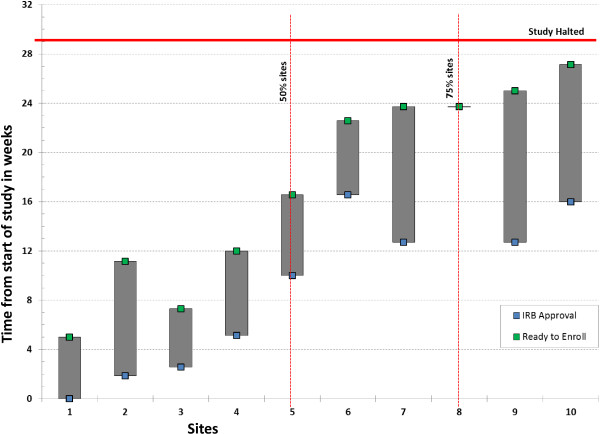
**Timeline of Institutional Review Board (IRB) approval and readiness to enroll patients at various sites.**

**Figure 3:**
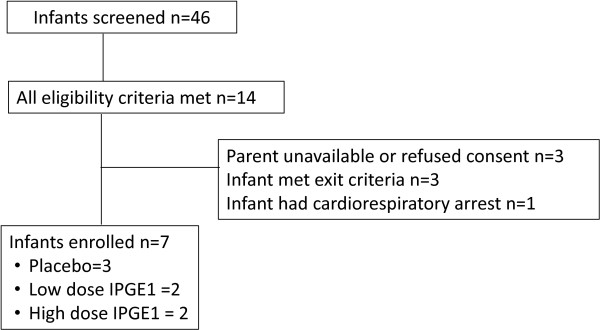
**Flow diagram of infants.**

The second pilot was undertaken because no infants could be enrolled in the first pilot; factors that hindered feasibility of the first pilot guided the study design of the second pilot. Detailed results for the second pilot RCT are discussed in the following. A total of 10 sites participated in the second pilot RCT. There was a lag from 33 to up to 90 days between IRB approval and site readiness to enroll patients due to the complexity of education regarding drug preparation and administration (Figure 2). Enrollment was halted for lack of feasibility after approximately six months; at that time, only seven patients had been enrolled.During the study period, 46 infants were screened at eight sites; 14 met eligibility criteria and seven were randomized (50%) (Figure 3). The reasons for non-enrollment were: parent unavailable or refused consent (three), met ECMO criteria (three), or cardiorespiratory arrest (one).

**Figure 4:**
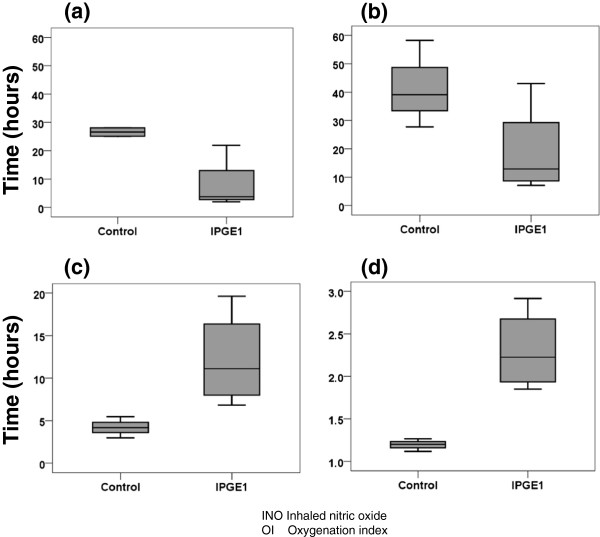
**Boxplots of timeline of events prior to initiation of study aerosol in control and combined IPGE**_**1**_ **groups (low-dose and high-dose IPGE**_**1**_ **).** INO, inhaled nitric oxide; OI, oxygenation index. **(a)** Age at INO start (hours; **(b)** Age at randomization (hours); **(c)** Interval from 1st OI > 15 and start of study aerosol (hours); **(d)** Interval from randomization to start of study aerosol (hours).

**Figure 5:**
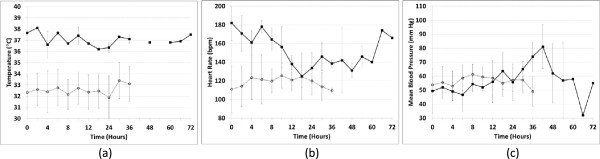
**Profile of temperature, heart rate, and mean blood pressure during study aerosol administration in control and combined IPGE**_**1**_ **groups (low-dose and high-dose IPGE**_**1**_ **).** Solid line with solid squares represents control subjects. Dotted line with open circles represents subjects receiving IPGE_1_ (combined data for low-dose and high-dose IPGE_1_). **(a)** Temperature; **(b)** Heart rate; **(c)** Mean blood pressure.

Of the seven infants randomized, three received aerosolized saline, and two each received high-dose and low-dose IPGE_1_. Aggregate data are presented for the control group and the two IPGE_1_ groups combined because of the small numbers of subjects in each group (Tables 1 and 2, Figures 4 and 5). The small numbers precluded formal statistical testing. Detailed individual patient data are presented in Table 3.

**Table 1:** **Delivery room characteristics of enrolled infants**

	Placebo n = 3	PGE _1_ n = 4
Maternal age mean, years (SD)	32.3 (6.8)	32.3 (9.4)
Outborn, n	3	3
Delivery by cesarean section, n	2	3
Birth weight mean, grams (SD)	3,590 (616)	3,425 (220)
Postmenstrual age mean, weeks (SD)	39.3 (1.5)	39.8 (0.5)
Apgar Score <3 at 1 minutes, n	0^a^	4
Apgar Score <3 at 5 minutes, n	0^a^	3
Intubation in the delivery room, n	0	4
Chest compressions in the delivery room, n	0	3

^a^Data missing for one subject. Outborn - born at an institution other than center where study intervention was administered.

**Table 2:** **Status at randomization**

	Placebo n = 3	PGE _1_ n = 4
Primary diagnosis (n)		
Idiopathic	2	0
Aspiration syndrome	1	4
Age at start of INO (hours) mean, (SD)	26.6 (2.1)^a^	7.9 (9.4)
Duration of INO before study aerosol (hours) mean, (SD)	8.0 (6.0)^a^	13.4 (7.2)
Interval between first OI ≥15 and study aerosol (hours) mean, (SD)	4.2 (1.2)	12.2 (5.6)
Interval between first and second qualifying blood gases (hours) mean, (SD)	2.4 (1.2)	4.7 (3.0)
Interval from randomization to study gas initiation (hours) mean, (SD)	1.2 (0.08)	2.3 (0.5)
Age at randomization (hours) mean, (SD)	41.7 (15.4)	19.0 (8.2)
OI on baseline ABG mean, (SD)	26.0 (9.5)	23.8 (17.3)
Therapies prior to randomization (n):		
Volume support	3	4
Vasopressor support	3	3
Sedation or analgesia	3	4
Neuromuscular blockade	2	3
Alkalosis	2	1
Surfactant	3	2

^a^Data missing for one subject. ABG, arterial blood gas; INO, inhaled nitric oxide; OI, oxygenation index.

**Table 3:** **Description of therapies prior to randomization, monitoring during aerosol administration, and outcomes of enrolled infants**

Case number	Before study aerosol				During study aerosol				After study aerosol		
	Surfactant	Inotropes	Pulmonary vasodilators	Ventilator Mode	Maximum Temperature (°C)	Maximum HR (bpm)	Minimum MBP (mmHg)	Duration of study aerosol (hours)	Was ECMO provided?	Time to ECMO (hours) ^c^	Neuroimaging at discharge
Control group:											
2	Yes	Dopamine	Milrinone Sildenafil	CMV	38.0	177	37	31.1	Yes	1.5	Normal study
4	Yes	Dopamine	Milrinone	HFO	37.5	185	32	72	No		Bilateral cephalhematomas, thinning corpus callosum
5	No	Dopamine Dobutamine	-	HFO	38.2	200	47	44.9	Yes	24.2	diffuse abnormality, white matter signal ↑ on T2 weighted MRI
Low-dose IPGE_1_ group:											
3	Yes	Dopamine Dobutamine	-	HFO	33.6	131	46	10.1	Yes	1.5	Normal study
6	No	-	Milrinone	HFO	30.6	118	43	36	Yes	Not known^a^	Unknown
High-dose IPGE_1_ group:											
1	Yes	Dopamine Dobutamine	-	HFO	33.5	142	45	39.5	Yes	1.2	↑ lactate peak suggestive of HIE
7^b^	Yes	Dopamine	-	CMV	33.7	121	63	34	Yes	4	Grade II IVH and posterior fossa hemorrhage

CMV, conventional mechanical ventilation; HFO, high frequency oscillator.; HIE, hypoxic ischemic encephalopathy; IVH, intraventricular hemorrhage; MRI, magnetic resonance imaging; ↑, increased.

^a^Infant received ECMO at another institution, time of ECMO not known.

^b^Infant received low-dose IPGE_1_ for first 24 hours though randomized to high-dose IPGE_1_; thereafter switched to low-dose IPGE_1_.

^c^Time from stop of study aerosol to initiation of ECMO (hours).

Delivery room characteristics of the enrolled infants in the control group and the two IPGE_1_ groups combined are presented in Table 1. Of note, all four infants assigned to low- or high-dose IPGE_1_ were intubated in the delivery room; three of these infants also received chest compressions. None of the infants in the control group were intubated or received chest compressions in the delivery room.

The underlying cause of NHRF was meconium aspiration syndrome in five infants and idiopathic in two infants; both infants with idiopathic NHRF were randomized to the control group (Table 2). Postnatal age at the start of INO ranged from two to 28 hours (Figure 4a). Duration of treatment with INO prior to start of study aerosol ranged from 3.5 to 23.5 hours, with the mean and median values being higher for infants in the combined IPGE_1_ groups. Infants were randomized to the study at an age range of 7.1 to 58.3 hours; all infants in the control group were >24 hours of age at time of randomization, whereas three of the four infants randomized to low- or high-dose IPGE_1_ were <16 hours of age (Figure 4b).

All infants had a trial of HFOV; five of the seven infants were receiving HFOV and two were receiving conventional mechanical ventilation at the time of randomization. All seven infants received fluid boluses prior to randomization, six infants received dopamine with or without dobutamine, and three infants received milrinone before randomization (Tables 2 and 3). Two infants in the control group received milrinone both before and during study aerosol administration; in one infant in the IPGE_1_ group, milrinone was discontinued 18 hours after study aerosol initiation. Only one infant received sildenafil before randomization; this infant belonged to the control group and received concurrent milrinone. All infants received sedation and/or analgesia, and five of the seven received neuromuscular blockade. Four of the seven infants received steroids before randomization.

All infants met the criterion of suboptimal response to INO. Data on the first OI ≥15 were available in six of the seven enrolled infants; four of the six infants had OI ≥29 (highest OI: 99). OI on the first qualifying arterial blood gas ranged from 16 to 69, with five of the seven infants having OI ≥25. OI on the second qualifying arterial blood gas ranged from 20 to 44, with six of the seven infants having OI ≥25. All seven infants had at least one documented OI >25, with six infants having OI >35 prior to starting the study aerosol. The interval between the first and second qualifying arterial blood gas ranged from one to nine hours. The time from randomization to study aerosol initiation ranged from 3.0 to 20.0 hours, with the interval being less than six in all infants in the control group and more than six hours in all infants in the combined IPGE_1_ groups (Figure 4c). The time from randomization to study aerosol initiation ranged from 1.1 to 2.9 hours, with all infants in the control group receiving study aerosol within 1.5 hours of randomization and all infants in the IPGE_1_ group receiving it after 1.5 hours (Figure 4d).

An echocardiogram was performed in all seven enrolled infants before study aerosol initiation. PDA was visualized in five infants, with direction of shunting being right to left or bidirectional in three, left to right in one, and no shunt documented in one.

Only one infant met the criteria for a complete response based on the one-hour ABG and this infant belonged to the control group. On the ABG obtained four hours after the start of the study aerosol, there was improvement in oxygenation in all three groups compared to the ABG obtained after 60 minutes (mean change in PaO_2_ being 30, 85, and 43 mmHg in control, low-dose, and high-dose IPGE_1_ groups, respectively). The duration of treatment with study aerosol ranged from 10 to 72 hours (Table 3). All seven enrolled infants survived. Six infants received ECMO; five of these received ECMO within 24 hours of discontinuation of the study aerosol. During study aerosol administration, acute deterioration was noted in three infants, two in the low-dose and one in the high-dose IPGE_1_ group, at 10, 36, and 34 hours of age, respectively. The first wean of the study aerosol had been attempted in two of these infants and was successful in one infant.

All enrolled infants were closely monitored for predefined adverse events. The lowest mean blood pressure recorded during study aerosol administration was 37, 43, and 45 mmHg in the control, low-dose, and high-dose IPGE_1_ groups, respectively (Table 3, Figure 5). The highest heart rate recorded during study aerosol administration was 200, 131, and 142 bpm in the control, low-dose, and high-dose IPGE_1_ groups, respectively. The highest temperature recorded during study aerosol administration was 38.2, 33.6, and 33.7°C in the control, low-dose, and high-dose IPGE_1_ groups, respectively. Two adverse events were reported during the study and nether was attributed to the study aerosol. One infant in the control group developed transient, self-resolved mild fever within five minutes of starting the study aerosol, which was completely resolved within six hours after onset. One infant in the control group developed moderate hypotension 66 hours after initiation of the study aerosol. It was completely resolved within an hour of its onset. All enrolled infants were carefully clinically monitored for signs of PDA after study aerosol discontinuation. None of the infants had clinical signs of failure secondary to PDA prompting an echocardiogram. One infant (in the high-dose IPGE_1_ group) developed seizures later in the hospital course, but the infant was not receiving study aerosol at that time. No protocol violations were reported during the study. There was one minor protocol deviation (ABG obtained >60 ± 15 minutes after aerosol initiation) and one pharmacy protocol violation (infant incorrectly received 150 ng/kg/min instead of the assigned 300 ng/kg/min dose of PGE_1_; this was recognized on the 2nd day of study aerosol administration).

## Discussion

We assessed the feasibility of two pilot RCTs to evaluate the use of two doses of IPGE_1_ in critically ill neonates with NHRF and were unable to recruit sufficient eligible subjects in the time period allowed. No infants were recruited in the first pilot. The second pilot was designed taking into account factors that hindered the feasibility of the first pilot. We demonstrated that continuous administration of aerosols was feasible in the second pilot. Of the 46 infants screened in this second pilot, only 14 met the eligibility criteria and seven were enrolled. Though the goal was to identify patients with OI ≥15, in fact all infants had much higher OIs at enrollment. All infants survived, and six of the seven received ECMO; five of these received ECMO within 24 hours of discontinuation of the study aerosol. There were no serious adverse events. Acute deterioration was documented in three infants in the IPGE_1_ groups 10 to 36 hours after start of study aerosol. There were systematic differences between the infants in the three groups; most notably, infants in both of the IPGE_1_ groups had lower Apgar scores, more need for delivery room resuscitation, an earlier age at presentation, and significantly lower temperatures throughout the study aerosol administration. The small sample size and clinical differences between the three groups precludes any inference regarding the efficacy of IPGE_1_ in PPHN.

The results of the two pilot RCTs reported in this study highlight the substantial investments of capital, human resources, technological expertise, and time in evaluating emerging new therapies [30]. Planning for these studies required performance of preclinical studies mandated by the United States Food and Drug Administration (FDA; addressing concerns about aerosol drug delivery with various modes of neonatal ventilation and/or INO; reaching consensus regarding patient management protocols, eligibility criteria, definition of meaningful outcome parameters, and use of nebulizer device in the study; development of study documents, training of site personnel, and obtaining IRB approvals.

The feasibility of conducting a large RCT was recognized to be an important limitation as several intervention clinical trials for NHRF were halted prematurely for lack of enrollment [22–25]; thus pilot studies were undertaken. Even though a survey of NRN sites between 2009 and 2010 suggested adequate numbers of patients eligible for this study, the pilot studies identified that the available patient population was very small. Foremost among factors contributing to lack of feasibility of this study was the decreased number of patients. This was attributed to improved perinatal care practices leading to decreased rates of both post-maturity and respiratory morbidity, as well as improved results in NHRF with the use of INO, surfactant, and HFOV [31, 32]. It is likely that with improved lung recruitment strategies, early use of surfactant and optimal delivery of INO, the current failure rate of INO may be considerably lower than that quoted in the literature [33]. Secondly, the consent process was challenging because of the short time window available, the high degree of stress parents’ experienced, frequent administration of medications that affect maternal cognition, and the lack of availability of parents when infants were transferred to tertiary care centers soon after birth for the management of PPHN.

The third factor that made these trials challenging was the novel nature of the study intervention. These pilot RCTs were the first trials of aerosolized selective pulmonary vasodilators in critically ill neonates with NHRF. The study protocol was complex and difficult to master for investigative teams. At the time of inception of this study, the MiniHeart low flow jet nebulizers were the only FDA approved nebulizers available for use in neonates; preliminary data were obtained using the MiniHeart nebulizer [26, 12, 34–38] and consequently the protocol allowed the use of MiniHeart nebulizers only in this study. Since then, ultrasonic and vibrating mesh nebulizers have been developed and are increasingly being used clinically [39, 40]. As there are advantages and disadvantages associated with each aerosol device, studies are needed to evaluate these devices for the delivery of IPGE_1_.

Another factor that contributed to the lack of feasibility of these trials was the long time lapse between IRB approval and initiation of enrollment. As defined by feasibility criteria in the second pilot, 75% of centers were IRB approved; however, readiness for study enrollment did not coincide with IRB approval. This may be due to the need for hospital approvals of research studies, as well as the need for training of site nurses, respiratory therapists, and pharmacists in implementing this novel intervention.

These studies highlight the challenges of performing clinical trials in critically ill neonates with hypoxemic respiratory failure. To date, only three interventions for NHRF (ECMO, INO, and HFOV) have been shown in large RCTs to significantly improve outcomes [4, 41, 42]. Objective evidence for other interventions for the treatment of term NHRF is lacking. Although INO has revolutionized outcomes, 40% of babies still do not respond, and thus further therapeutic improvements are critical. Other drugs, including sildenafil, inhaled iloprost, endothelin antagonists, and antioxidants, are actively being explored for the treatment of NHRF, but no large RCTs in critically ill neonates have been reported. A failure to recruit patients in this trial has important implications for other clinical trials in this patient population. Inability to design definitive clinical trials to evaluate newer therapies in NHRF will propagate the practice of ‘off-label’ use of drugs that have been approved for use in adults first [43]. From developmental and metabolic standpoints, the disease processes in children and their responses to therapies are very different from those in adults [44]. The search for robust evidence to guide the safe therapy of children and neonates with pulmonary hypertensive vascular disease is crucial.

Important lessons can be learned from clinical trials in adult and pediatric pulmonary hypertension where similar challenges with recruitment are encountered. The prevalence of pulmonary hypertension in adults, children, and neonates is insufficient to support formal examination of all potential drug targets [45]. Recognizing the need for innovative approaches [43, 46, 47], a workshop was established to consider alternative clinical trial designs in pulmonary hypertension, in order to raise the likelihood of new treatments reaching patients. Acknowledging that classical sample size calculations often lead to unrealistic expectations of the number of subjects to be recruited, Offringa and van der Lee described statistical and epidemiological aspects of small sample approaches and emphasized the importance of maximizing information obtained from the few subjects enrolled [48]. Development of national and international databases with common entry criteria may be an alternative approach. Clinical studies in pulmonary hypertension might be enhanced by a consortium approach that utilizes the expertise of academic medicine, the treatment initiatives of the pharmaceutical industry, and study design from funding agencies interested in biological mechanisms [49]. To address this challenge, the Pulmonary Hypertension Academic Research Consortium was created as a forum to openly discuss strategies for clinical trials in pulmonary hypertension that would benefit all of the stakeholders. The goals of the consortium were to establish consensus clinical endpoint definitions for future clinical trials, advance the conduct of clinical research in the field, identify modern strategies for clinical trials, and provide guidance to the pharmaceutical industry to allow them to better identify and develop treatments with the most promise. Similar approaches need to be explored in NHRF.

## Conclusions

In conclusion, there are important lessons to be learned from the two pilot RCTs described in this report that will be instrumental in designing future studies in this critically ill population. The second pilot demonstrates that it is possible to administer aerosols continuously in the Neonatal Intensive Care Unit for extended periods of time. When defining feasibility criteria, it needs to be recognized that readiness to commence study enrollment does not coincide with IRB approval. This lag needs to be adjusted for in future clinical trials. In this small sample size, there were no serious adverse events reported with the two doses of IPGE_1_; future studies should explore the efficacy of the higher dose and consider dose escalation to identify the optimal therapeutic dose. The second pilot also demonstrates that though the goal was to identify patients with OI ≥15, in fact all the babies had much higher OIs at enrollment as has been shown in other RCTs in NHRF [4]. This suggests that future trials randomize infants at an even earlier stage, preferably at the time of initiation of INO. Another approach would be to randomize infants to placebo and treatments soon after PPHN is diagnosed, and then add specific therapy, such as INO. Future studies should allow the use of nebulizer devices approved by the FDA for use in neonates with provision for a subgroup analysis by the nebulizer device during the evaluation of data. Ventilator strategy, use of supportive therapies (such as surfactant and inotropes), and underlying cause of PPHN should be taken into account while designing future studies. Both of the pilot trials reinforce the challenges of performing RCTs in this critically ill population of patients and the need for pragmatic study designs reflective of the real-world situations with a realistic timeline.

## Electronic supplementary material

Additional file 1: **List of approving Ethical Committees.** (DOCX 16 KB)

## Abbreviations

ABG: Arterial blood gas
CNV: Conventional ventilation
DSMC: Data Safety Monitoring Committee
FiO2: fractional inspired oxygen concentration
HFOV: High frequency oscillatory ventilation
INO: Inhaled nitric oxide
IPGE_1_: Inhaled Prostaglandin E_1_
IRB: Institutional review board
MAP: mean airway pressure)
NHRF: Neonates with hypoxemic respiratory failure
NICHD: National Institute of Child Health and Human Development
NICU: Neonatal intensive care unit
NRN: Neonatal Research Network
OI: oxygenation index
P_a_O2: arterial oxygen tension
PGE_1_: Prostaglandins E_1_
PGI_2_: Prostaglandins I_2_
PPHN: Persistent pulmonary hypertension of the newborn
RCT: Randomized control trial
SPO_2_: Pulse oximeter O_2_ saturation.
