# Supplementary material for: Inhaled PGE1 in neonates with hypoxemic respiratory failure: two pilot feasibility randomized clinical trials
Source: Trials. 2014 Dec 12;15:486. doi: 10.1186/1745-6215-15-486 (PMC4414424; doi:10.1186/1745-6215-15-486)
Supplement: Supplementary file 1 — Additional file 1: List of approving Ethical Committees. (DOCX 16 KB) [file 13063_2014_2415_MOESM1_ESM.docx]

**Additional file 1**

**File 1: Ethical Boards**:

- University of Alabama at Birmingham (IRB00000196, IRB00000726)
  - - Women & Infants hospital (Brown University) (IRB11-0099)
    - University at Buffalo, State University of New York, Children & Youth IRB (IRB00004088)
    - Parkland Health & Hospital System (University of Texas Southwestern) (IRB #092011-003)
    - Duke University Health System IRB (Pro00000011, Pro00033527)
    - Stanford University (IRB 3467 SQL 96548)
    - Tufts University (IRB#8392)
    - University of Utah (IRB# 00027525)
    - University of Iowa (IRB# 200801701, IRB# 201111721)
    - Nationwide Children's (FWA00002860, IRB11-00683)
    - University of New Mexico Health Sciences Center (HRRC #08-100, HRPO #11-523)
    - University of California, Los Angeles, (FWA00004642, IRB00000172)
    - Wayne State University (IRB086005MP4F, IRB082111MP2F)
    - RTI IRB (FWA 3331)

Please note that although the site has IRB approval not all sites were issued an approval number.
